# An Exploratory Search for Potential Molecular Targets Responsive to the Probiotic *Lactobacillus salivarius* PS2 in Women With Mastitis: Gene Expression Profiling vs. Interindividual Variability

**DOI:** 10.3389/fmicb.2018.02166

**Published:** 2018-09-13

**Authors:** Javier de Andrés, Esther Jiménez, Irene Espinosa-Martos, Juan Miguel Rodríguez, María-Teresa García-Conesa

**Affiliations:** ^1^ProbiSearch, SLU, Madrid, Spain; ^2^Department of Nutrition, Food Science and Technology, University Complutense of Madrid, Madrid, Spain; ^3^Research Group on Quality, Safety and Bioactivity of Plant Foods, Department of Food Science and Technology, Centro de Edafología y Biología Aplicada del Segura-Consejo Superior de Investigaciones Científicas, Murcia, Spain

**Keywords:** microarrays, transcriptomics, RT-qPCR, molecular biomarkers, mastitis, human intervention trials

## Abstract

Probiotics constitute an attractive alternative in the battle against microbial infections. Oral administration of certain strains of lactobacilli isolated from human milk has resulted in an effective reduction of the bacterial load as well as an improvement of the mastitis-associated symptoms. Nevertheless, little is yet known about the potential molecular mechanisms and specific targets implicated in these effects. Transcriptomic profiling has been used to search for disease-associated and therapy-responsive molecules in different disorders and experimental models. We have applied for the first time a gene expression-based molecular approach to explore for potential targets responsive to intervention with a probiotic in: (i) breast milk somatic cells (*n* = 17) and (ii) blood leukocytes (*n* = 19). Women with mastitis ingested a new strain of lactobacilli, *Lactobacillus salivarius* PS2 (3 × capsules per day, each capsule contained ~9.5 log10 CFU) for 21 days. We applied Affymetrix microarrays and Taqman one-step quantitative reverse transcription PCR (RT-qPCR) to analyze and compare gene expression changes between samples pre- and post-treatment. Our results substantiate the involvement of inflammatory and cell-growth related pathways and genes in the breast milk somatic cells following the intake of *L. salivarius* PS2. Individual analyses of selected genes: (1) supported the upregulation of *STC1* and *IL19* and the downregulation of *PLAUR* and *IFNGR1* in the somatic cells of the patients as potential targets responsive to the probiotic, (2) detected a lack of a relationship between the gene expression responses in the two types of cells, and (3) evidenced a substantial interindividual variability in the gene expression changes in both types of cells. Our study provides an insight into the essentiality of incorporating the study of tissue-specific interindividual molecular responsivity into future clinical intervention trials to further understand the complexity of human gene expression responses to therapy and the potentiality of selecting appropriate responsive targets.

## Introduction

Human milk is a complex biological fluid produced by the mammary gland and adapted to satisfy the requirements of the newborn at different growth stages. Human milk also educates the infant immune system, enhancing oral tolerance, and confers a certain degree of protection against pathogens (Andreas et al., 2015; Goldsmith et al., 2015; Boix-Amorós et al., 2016). Colostrum and milk are continuous sources of commensal, mutualistic and potentially beneficial bacteria to the infant gut (Gueimonde et al., 2007; Collado et al., 2009; Martín et al., 2009; Hunt et al., 2011). Among the complex bacteria community present in the human milk of healthy women, staphylococci and streptococci are the dominant genera although present at a low concentration (Martín et al., 2003; Fernández et al., 2013; Avershina et al., 2018). Mastitis is an inflammation of the mammary gland in response to infection usually caused by bacteria. It constitutes a major problem experienced by breastfeeding women, with incidences rates of around 20% in developed countries but with wide variations among studies (Khanal et al., 2015). Fresh milk samples from women suffering mastitis have been shown by culture techniques to contain a high amount of staphylococci and (or) streptococci as well as a dramatic decrease of bacterial diversity (Mediano et al., 2017).

To treat this infection, physicians generally use antibiotics although, it is now well established that antibiotics pose several critical problems, e.g., the attainment of antibiotic resistance by resident bacteria and the development of gastrointestinal disorders in the host (Ferrer et al., 2017; Iizumi et al., 2017; Hwang et al., 2018). As a potential alternative to antibiotic treatment, some studies have explored the use of different probiotic strains to combat infectious mastitis (Jiménez et al., 2008; Arroyo et al., 2010). In these articles, the authors have shown a significant reduction in the milk bacterial counts and a rapid improvement of the mastitis symptoms following oral intake of different species of lactobacilli, i.e., *L. salivarius* CECT5713 plus *L. gasseri* CECT5714 (Jiménez et al., 2008), and *L. salivarius* CECT5713 or *L. fermentum* CECT5716 (Arroyo et al., 2010). The presence of these strains in the human milk of the mastitis-suffering women at the end of the study and the observed anti-inflammatory response of the patients support a true probiotic effect for these lactobacilli (Jiménez et al., 2008). More recently, the *L. salivarius* PS2, a probiotic strain isolated from the human milk of healthy women, was tested against mastitis in a clinical trial. The study was designed to search for microbiological, biochemical and immunological biomarkers in response to the treatment with this probiotic. At the end of the trial, the patients that ingested *L. salivarius* PS2 experienced a general improvement of their condition. In addition, the mammary gland microbiota balance was restored (Espinosa-Martos et al., 2016).

The complex cellular and molecular mechanisms underlying the response triggered in the mammary gland to the pathogens are not yet fully understood. Invading microorganisms responsible for the mastitis activate a complex immune defense process that involves resident and recruited immune cells, endothelial cells and epithelial mammary cells resulting in an increase in the number of somatic cells (SC) in the milk (Cremonesi et al., 2012). Concerning the molecular responses, over the past years, transcriptomic studies using microarrays or RNA sequencing have been repeatedly applied to identify genes and pathways altered in the mammary tissue in response to infection by pathogenic bacteria such as *Staphylococcus aureus* or *Streptococcus uberis* using *in vitro* and animal studies (Yang et al., 2008; Swanson et al., 2009; Richards et al., 2013; Günther et al., 2017; Kosciuczuk et al., 2017; Petzl et al., 2018). A recent study has described, for the first time, the milk cell transcriptome across the lactation cycle and during mastitis infection in women (Sharp et al., 2016). However, there were no studies investigating the molecular response of the human mammary gland to an intervention with a probiotic.

The main purpose of this study was to explore the feasibility of using microarray and RT-qPCR techniques to search for specific molecules as new targets responsive to the oral intake of the probiotic *L. salivarius* PS2 in the human mammary gland affected with mastitis infection. To achieve this objective, we have investigated: (i) gene expression changes in breast milk isolated SC as a source of mammary tissue obtained from women with mastitis before and after intervention with the probiotic, and (ii) the potential relationship between some of the significantly altered genes in the SC with those occurring in peripheral blood leukocytes obtained from the same patients. The present study also examines the interindividual variability in tissue gene expression responses and highlights the relevance of further investigating and understanding this critical issue in order to improve our efficacy in the selection of molecular biomarkers of response.

## Materials and methods

### Subjects and study design

This study is part of the trial NCT01124448 registered at the ClinicalTrials.gov database. Full details can be found in a previous report (Espinosa-Martos et al., 2016). Briefly, a total of 31 women, 23 patients with mastitis symptoms and 8 without any symptom of the disease (healthy group) participated in the study. All volunteers gave written informed consent to the protocol (reference 10/017E) approved by the Ethical Committee of Clinical Research of the Hospital San Carlos (Madrid, Spain). A questionnaire about their general health status, pregnancy development, delivery, lactating period and medication taken was filled out by all the participants. The demographic characteristics of the women are summarized in Supplementary Table S1. The criteria for selecting the participants were described elsewhere (Espinosa-Martos et al., 2016). None of the participants ingested any commercial probiotic supplements during the intervention. Based on the evidence from previous clinical trials (Jiménez et al., 2008; Arroyo et al., 2010; Vázquez-Fresno et al., 2014), the Ethical Committee did not allowed a control group of women with mastitis (either left untreated or receiving a placebo).

**Figure 1:**
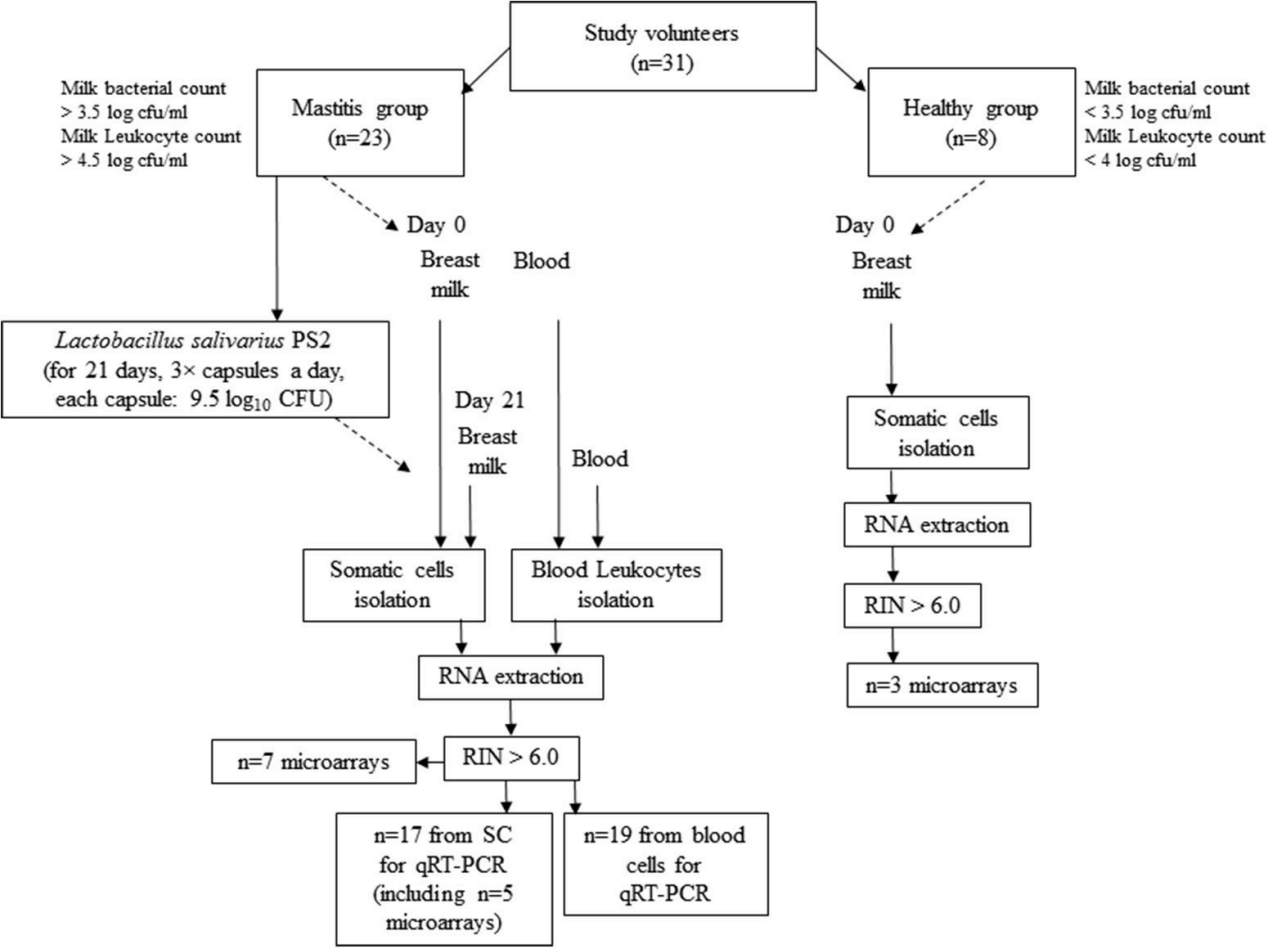
Diagram representing the work flow of the study and experimental protocol.

The intervention period was 21 days based on previous studies that attained good results with other probiotics in a similar period of time (Arroyo et al., 2010). During this period the volunteers with mastitis symptoms consumed 3 × capsules per day, each of them containing ~50 mg of freeze-dried powdered *L. salivarius* PS2 ~9.5 log10 CFU). The capsules were manufactured by Biopolis (Valencia, Spain) and preserved at 4°C throughout the study. Samples of breast milk and blood were taken at day 0 and day 21 of the intervention and further processed for cell isolation. A diagram summarizing the study design and protocol is shown in Figure 1.

### Breast-milk somatic and blood cells isolation

Milk samples were collected at baseline (both from patients with mastitis and healthy donors) and at the end (patients with mastitis) of the intervention following the protocol previously described (Espinosa-Martos et al., 2016). Milk total somatic cells (SC) were isolated from 10 mL of fresh milk after centrifugation (620 × *g*, 20 min) and removal of fat layer and supernatant.

Leukocytes (mononuclear cells and granulocytes) were also isolated from women with mastitis at the beginning (day 0) and at the end of the intervention period (day 21). Volunteers were instructed to fast overnight before each blood collection. Blood samples were collected in heparinized tubes (BD Vacutainer, Franklin Lakes, NJ, USA) between 8 and 10 a.m. to minimize circadian variations and processed within 2 h after extraction. Leukocytes were isolated by gradient centrifugation as follows: 6 mL of blood was diluted (1:1) with RPMI 1,640 + 5% of bovine fetal serum (Gibco) cell culture medium and centrifuged by density gradient with Histopaque-1077 and Histopaque-1119 (Sigma–Aldrich, Madrid, Spain) according to the manufacturer instructions. After washing the cells (2 ×) with RPMI 1640 + 5% of bovine fetal serum, Allprotect Tissue Reagent (QIAGEN) was added for the preservation of the RNA and proteins. All samples were stored at −80°C.

### RNA extraction and purity

Total RNA was isolated from all samples using the AllPrep DNA/RNA/Protein Mini Kit (Qiagen, Madrid, Spain) following the manufacturer recommendations. RNA concentration and purity were checked using the Nanodrop ND-1000 UV-Vis Spectrophotometer (Nanodrop Technologies) and the Agilent 2100 Bioanalyzer (Agilent, Madrid, Spain). Only those samples with a ratio Abs_260_/Abs_280_ between 1.8 and 2.1 and RNA integrity number (RIN) values above 6.0 were used for gene expression analyses. From the initial number of participants, 7 (women with mastitis) and 3 (healthy women) milk-derived SC samples were used for microarrays. A total of 17 samples (women with mastitis, including 5 of the samples already used for microarrays) were then used for RT-qPCR. Regarding the blood leukocytes, we collected 19 RNA high-quality samples (women with mastitis) for RT-PCR (Figure 1). Purified RNA samples were divided in aliquots and kept at −80°C until further analysis by microarrays and (or) RT-qPCR.

### Expression profiling using microarrays

RNA from the breast milk isolated SC was hybridized onto the GeneChip® PrimeView™ Human Gene Expression Array (Affymetrix, CA, USA) that contains a total of 49,395 probe sets. Microarrays were carried out following the manufacturer's instructions and GeneChips were scanned using the GeneChip Scanner 3000 7G with an autoloader. Emission intensities of the different probe sets were captured and transformed (.CEL files) by Affymetrix GeneChip® Command Console® Software (Affymetrix, CA, USA). Probes were summarized to gene-level data, background subtracted, and expression values normalized to log base-2 in Partek Genomics Suite v6.6 (Partek, St. Louis, MO) using the Robust MultiChip Average (RMA) algorithm and the.CEL files (Bolstad et al., 2003). Significant statistical differences (*p*-values) in gene expression fold-changes (FC) were generated with Partek Genomics Suite 6.6 using the ANOVA test. Probes were considered to be significantly and differentially expressed if the *p*-value was < 0.05 and had minimum FC-value < −1.2 and >+1.2. Minimum Information About a Microarray Experiment (MIAME) compliant data have been deposited in NCBI's Gene Expression Omnibus database (GEO; http://www.ncbi.nlm.nih.gov/geo/) at the accession number GSE65152.

### Microarray data analyses

Hierarchical clustering and principal component analyses (PCA) of microarray results (genes with significantly altered expression) were done using the Partek Genomics Suite® 6.6. The lists of significant genes were also used for functional analysis using Gene Ontology in the DAVID platform (Huang da et al., 2009), and KEGG pathways enrichment analysis using Partek Pathway™ and the Fisher's exact test.

### RT-qPCR analyses of target genes

One-step quantitative reverse transcription PCR (RT-qPCR) (TaqMan system, Applied Biosystems, ABI, Madrid, Spain) was applied to validate microarray results and further measure differential expression in RNA isolated from milk SC (*n* = 17) and from blood cells (*n* = 19) attained from patients with mastitis. The analyses were carried out at the Science Park of the Cantoblanco Campus (Madrid, Spain) and were run on an ABI 7500 system following the manufacturer's protocol. We used a total reaction volume of 25 μL in a MicroAmp Optical 96-well plate covered by optical adhesive covers and TaqMan Universal Master Mix (ABI, Madrid, Spain). All assays were undertaken at the same time under identical conditions and in triplicate. Supplementary Table S2 includes the list of the subset of genes analyzed (*STC1, PLAUR, IFNGR1, VASP, IL19, IFNA1, DCD*), the reference genes used in the analysis (*GAPDH, TBP, POLR2A*) and the corresponding ABI references of the TaqMan Assays used for validation.

NormFinder algorithm (Andersen et al., 2004) was implemented to determine the stability and ranking of expression of the endogenous control genes. The estimated values for *GAPDH, TBP*, and *POLR2A* indicated a good stability for the three genes. We additionally calculated the coefficient of variation [CV (%)] of the Ct values for each reference gene across all the samples, 4.32, 4.41, and 5.46%, respectively. The average Ct values (three technical replicates) for targeted and reference genes were obtained from each reaction to determine the occurrence of gene expression in the tested samples. Samples with a Ct value ≥35 were considered not detected. The relative mRNA level of each targeted gene was calculated by the 2^−ΔΔ*Ct*^ method (Livak and Schmittgen, 2001). Final data are expressed as the mean FC-value normalized against the three selected reference genes. Genes were considered to be upregulated if FC >+1.2 and downregulated if FC < −1.2. The median of the sample population and the range [Min, Max] values are indicated.

Potential associations between gene expression changes in the milk SC and the leukocytes and between gene expression changes and effects of the probiotic in the bacterial counts, immune cell counts and in the levels of several specific plasma cytokines were examined using the Spearman's rank correlation coefficient. Individual data for bacterial counts, immune cell counts and plasma cytokines were obtained from a previous publication (Espinosa-Martos et al., 2016).

## Results

### Transcriptomic profiling and functional analysis

Good quality RNA samples obtained from the human milk SC isolated from 7 women with mastitis and 3 healthy volunteers, collected at day 0 and day 21 post-treatment with the probiotic, were used for microarray analyses. We first compared the gene expression levels between patients with mastitis and healthy volunteers at baseline (day 0), i.e., differentially expressed genes potentially associated with the disease condition. A total of 1,377 probes were identified as significantly changed (*p*-value < 0.05) of which 309 were upregulated and 1,068 were downregulated (Supplementary Table S3). Next, we compared patients with mastitis at day 21 post-intervention vs. day 0, i.e., differentially expressed genes possibly responsive to the consumption of the probiotic in the mastitis condition. A total of 387 probes were found upregulated and 81 downregulated (*p*-value < 0.05; Supplementary Table S4).

**Figure 2:**
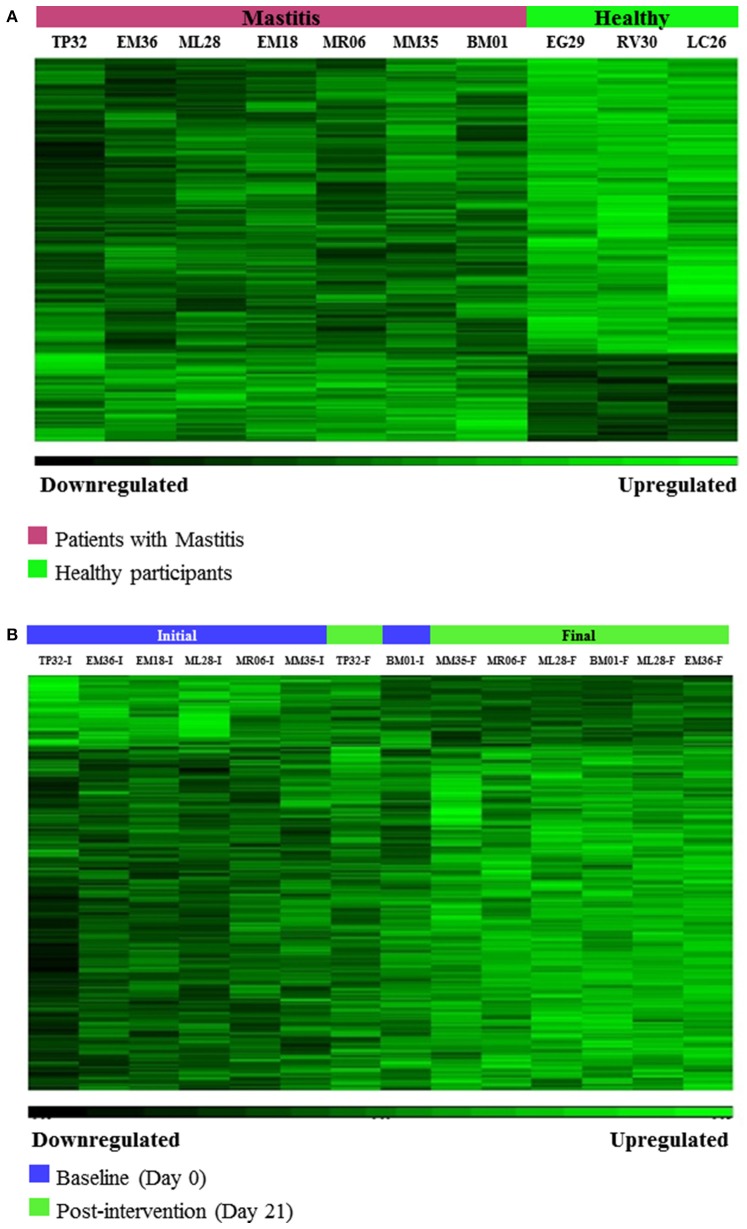
**(A)** Hierarchical Clustering of microarrays results: Gene differences associated with the disease, i.e., differentially expressed genes between patients with mastitis and healthy volunteers at baseline (day 0), (*p*-value < 0.05, FC > 1.2 or < -1.2). **(B)** Hierarchical Clustering of microarrays results: Gene changes associated with the response to the intervention with *L. salivarius* PS2 in patients with mastitis, i.e., differentially expressed genes between patients with mastitis after 21 day-treatment with the probiotic *vs*. patients with mastitis at baseline (day 0), (*p*-value < 0.05, FC > 1.2 or < −1.2).

**Figure 3:**
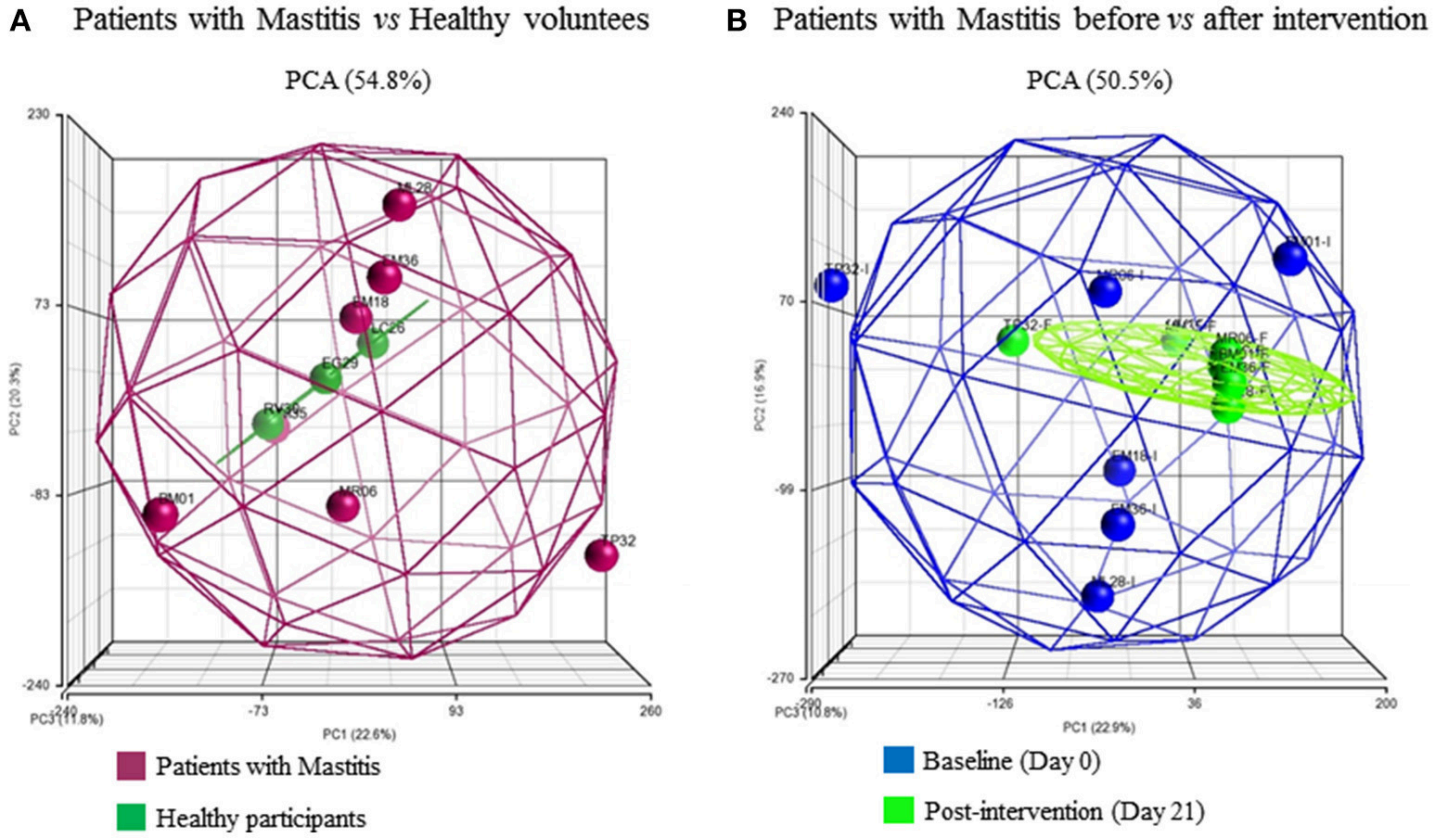
Three-dimensional representation according to principle component analysis (PCA) of the microarray differential gene expression significant data. Spheroids collect the at least 95% of the samples in each comparison and each sphere represents a patient: **(A)** patients at baseline, Mastitis *vs* Healthy groups, **(B)** Mastitis patients at day 0 vs. day 21 post-intervention with the probiotic. The color of the patients represents their health status or time-point respectively.

Hierarchical clustering was used to group individuals on the basis of their similarity in the pattern of the significant changing genes for the two pairs of comparisons (Figures 2A,B). This clustering provided an overview of the molecular differences that existed between the patients with mastitis and the healthy participants, i.e., genes potentially associated with the disease (Figure 2A). It also showed the differences in the mastitis patients before and after treatment with the *L. salivarius* PS2 probiotic for 21 days (Figure 2B). This analysis was also giving a first glance at the variation in gene expression across the individuals. Discriminant PCA (Figure 3) was also performed to further visualize the differences between the patients with mastitis vs. the healthy volunteers at baseline (Figure 3A) and the patients before and after the intervention with the probiotic (Figure 3B). The PCA analyses identified three components accounting for ~55% of the total variability for the comparison between women with mastitis and healthy women and ~50% for the comparison between women with mastitis before and after intervention. This analysis supported an initial scattering of the patients with mastitis (e.g., high interindividual variability) and the posterior coming closer of these patients in response to the treatment with the probiotic suggesting that the intake of the probiotic might be associated with a shared molecular response in the milk SC of the women with mastitis.

Uppermost enriched Gene Ontology terms and altered inflammation- and cell growth/survival-related pathways among differentially expressed genes between patients with mastitis and healthy individuals and, in patients following treatment with the probiotic are listed in Supplementary Table S5. Among the genes changing in the volunteers with mastitis in comparison with the healthy participants (potentially disease-related deregulated processes), some of the most enriched Gene Ontology terms associated with upregulation were immune response and activation of immune cells (lymphocytes, T-cells, natural killer cell activation process), indicative of the triggering of the defense mechanisms against the infection. Alteration of “cell proliferation, adhesion and angiogenesis” as well as general cell processes such as “transmembrane receptor tyrosine kinase mediated signaling pathway” were also detected as upregulated by the analysis. The downregulated clustering indicated the involvement of “mRNA processing,” “glutamine metabolism,” and “membrane associated processes” in the SC from patients with mastitis in comparison with healthy cells. Treatment with the probiotic in the participants with mastitis also revealed alteration of “cell death,” “transcription,” “amino acid metabolism,” “transmembrane transport” and the “glycosylphosphatidylinositol (GPI) anchor biosynthesis,” a molecule that attaches some membrane proteins to the lipid bilayer of the cell membrane.

**Figure 4:**
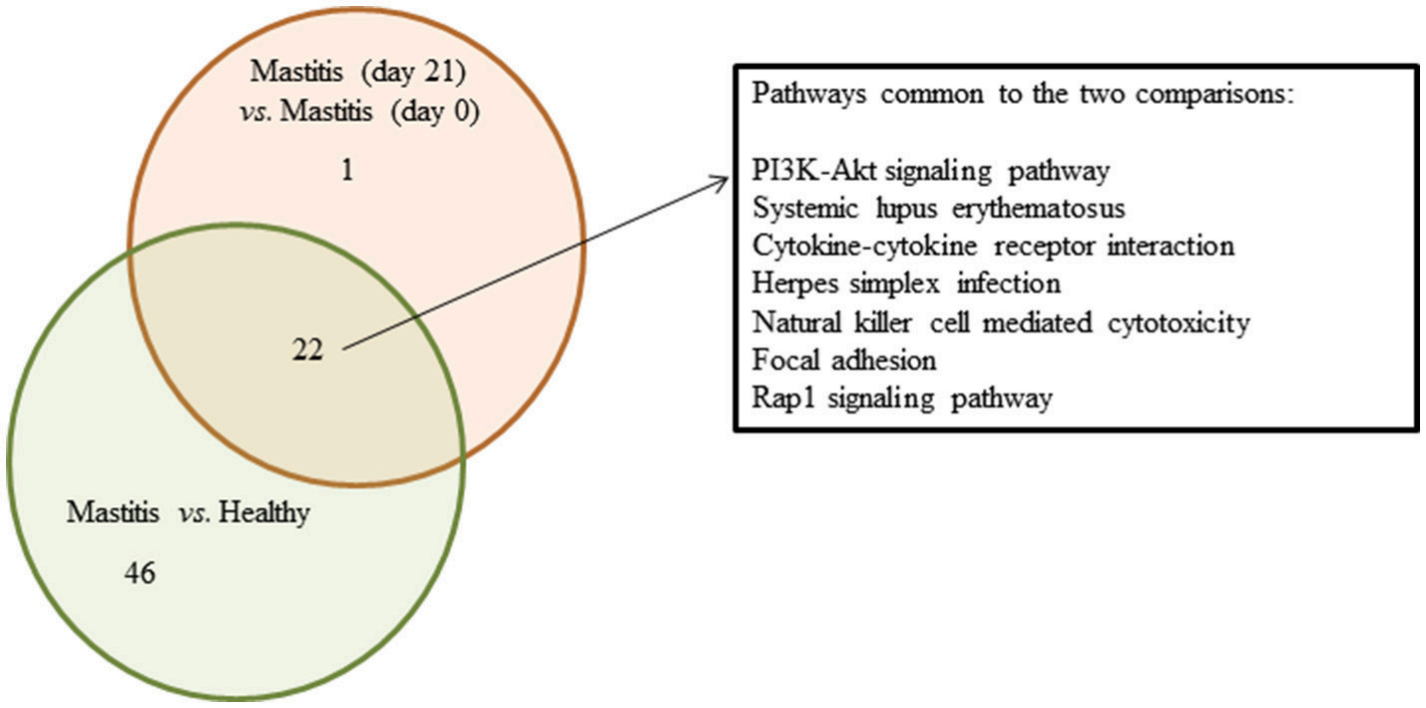
Venn diagram summarizing overlapped pathways related to infection/immune diseases and (or) responses among the DE probes detected in SC of women with mastitis and healthy participants, and women with mastitis before and after the treatment with the probiotic. Green circle shows the number of pathways obtained when comparing samples of women with mastitis and healthy volunteers at day 0; red circle shows the number of pathways obtained when comparing women with mastitis at day 0 and at day 21. Intersection represents the number of overlapped pathways. In the separate square we include the top regulated common pathways.

The Partek analysis suite confirmed the involvement of inflammation-related responses, i.e., “natural killer cell mediated cytotoxicity,” “chemokine signaling pathways,” “cytokine-cytokine receptor interaction” as well as other infection and immune disease-related processes in the mastitis disease and in the response to the intervention with the probiotic. Several specific signaling pathways were additionally identified, the “PI3K-Akt,” the “Jak-STAT” or the “Rap1.” A Venn diagram illustrating the top represented and overlapped pathways related to inflammation responses among the differentially expressed probes as detected by the Partek analysis in the two comparisons is depicted in Figure 4. Various specific identifiers commonly found in the two cases are marked out in a separate box: “PI3K-Akt signaling pathway,” “systemic lupus erythematosus,” “cytokine-cytokine receptor interaction,” “herpes simplex infection,” “natural killer cell mediated cytotoxicity,” “focal adhesion” and “Rap1 signaling pathway.”

### RT-qPCR analyses of selected gene targets in the breast milk isolated SC and in the blood mononuclear isolated cells from women with mastitis

The genes selected for validation and further analysis using qRT-PCR are listed in Table 1. The number of probes representing the gene in the array, their corresponding FC (median values, *n* = 7) and the significance of the change (*p*-values) are also specified. These genes were found to exhibit significant changes in the milk SC of women with mastitis after the treatment with the probiotic. To further support the biological interest and selection of these genes as potential molecular targets associated with mastitis, Table 1 also compiles some of the main biological functions related with mastitis, inflammation and (or) breast tissue disease in which these genes have been reported to be involved.

**Table 1:** Genes selected for validation and further analysis by RT-qPCR.

**Gene**	**Changes (median, *n* = 7) by microarrays: number of probes represented in the array and corresponding change (FC, significance *p*-value)**	**Gene ontology**	**Description**
Symbol	Mastitis (day 21) vs. Mastitis (day 0)	Biological process	Mastitis/inflammation/cancer/ Breast or mammary tissue
*STC1*	2 probes ↑ (+1.8, *p =* 0.0497) ↑(+1.7, *p =* 0.0469) 2 probes ↑(+1.8, NS) ↑ (+1.7, NS)	Cellular calcium ion homeostasis/ cell surface receptor linked signaling pathway/ response to nutrient	Endocrine factor with potential local action in Ca(2+) and P(i) transport; effect on the sub-cellular functions of mitochondria and endoplasmic reticulum responding to oxidative stress and unfolded protein response (Yeung et al., 2012). Secreted glycoprotein implicated in several pathologies including angiogenesis and inflammation. Aberrant expression has been reported in breast cancer (Chang et al., 2015).
*PLAUR (uPAR)*	1 probe ↓(−2.0, *p =* 0.0399) 4 probes ↓(−2.0, NS) ↓(−1.9, NS) ↓(−1.9, NS) ↓ (−1.4, NS)	Chemotaxis/signal transduction/ blood coagulation	Elevated levels of PLAUR found in aggressive breast cancer. Anti-uPAR antibodies were effective at reducing breast tumor burden *in vivo* (LeBeau et al., 2013). Central mediator of growth factor-induced endothelial cell migration and angiogenesis in breast cancer (Poettler et al., 2012). The plasminogen activation system has a key role in the regulation of all the phases of the inflammation process (Del Rosso et al., 2008). Induced in udder tissue of heifers inoculated with *Streptococcus uberis* (de Greeff et al., 2013).
*IFNGR1*	1 probe ↓(−1.5, *p =* 0.0499) 1 probe ↓ (−1.6, NS)	Signal transduction/blood coagulation/response to virus	Low expression of IFNGR1 leads to a functional blockade of IFNγ signaling and is associated with breast cancer (Chen et al., 2012). Antigen-specific inflammation in cows' mammary gland is characterized by overexpression of interleukins and IFN-γ (Rainard et al., 2013).
*VASP*	1 probe ↓ (−1.4, *p =* 0.0187)	Neural tube closure/ actin cytoskeleton organization	Suppression of VASP is associated with TNF-α and HIF-1α induced inhibition of breast cancer cell adhesion and proliferation (Su et al., 2012). Downregulation of VASP expression inhibits breast cancer cell migration and invasion (Zhang et al., 2009).
*IL19*	1 probe ↑(+1.5, *p =* 0.0460) 1 probe ↑ (+1.3, NS)	Oxygen and reactive oxygen species metabolic process/ immune response/apoptosis/ signal transduction/ interleukin-6 biosynthetic process	IL-19 is produced by monocytes, and activates these cells in an autocrine and paracrine fashion to release the cytokines IL-6, TNF-α, and numerous ROS. Monocyte IL-19 production is downregulated by IFN-γ (Commins et al., 2008). IL-19 has an autocrine effect in breast cancer cells; it promotes cell proliferation and migration (Chen et al., 2013).
*IFNA1/13*	1 probe ↑ (+1.3, *p =* 0.0132)	Defense response/response to virus	Most tissues and cell types produce IFN-1 when exposed to appropriate pathogen or danger-associated molecular patterns (Rauch et al., 2013). IFN-α1 gene transfer in *in vivo* models of breast cancer induced neovascularization and tumor growth. Gene therapy with IFN-α1 might be useful for treatment of breast cancer (Indraccolo et al., 2002).
*DCD*	1 probe ↑ (+1.3, *p =* 0.00049)	Killing of cells of another organism/ defense response to bacterium	Dermcidin participates in the host defense against *Staphylococcus aureus* skin infection (Ryu et al., 2014). Dermcidin expression is associated with breast cancer progression (Brauer et al., 2014).

*These genes were found to exhibit some significant (p < 0.05) changes by microarrays in breast milk SC from women with mastitis (n = 7) after the intake of the probiotic (L. salivarius PS2). We have indicated the results of all the probes represented in the array for each gene with their corresponding change and significance. We have also included functional information about these genes based on Gene Ontology and biological processes associated with breast tissue diseases based on the literature. SC, Somatic cells; FC, Fold-change; STC1, Stanniocalcin 1; PLAUR(uPAR), Plasminogen activator, urokinase receptor; IFNGR1, Interferon gamma receptor 1; VASP, Vasodilator-stimulated phosphoprotein; IL19, Interleukin-19; IFNA1/13, Interferon alpha-1/13; DCD, Dermcidin; IFNγ, Interferon gamma; TNF-α, Tumor necrosis alpha; HIF-1α, Hypoxia Inducible Factor 1 Alpha Subunit; IL-19, Interleukin 19; IL-6, Interleukin 6; ROS, Reactive oxygen species. ↑, Upregulation, ↓, Downregulation; NS, Not significant*.

Validation of the microarray analyses by Rt-qPCR was carried out in five of the samples used for microarrays and the results were all confirmed for *STC1, PLAUR, IFNGR1, VASP*, and *IL19* (Table 2). Next, we analyzed the expression of these genes in the SC isolated from other patients included in the study. A summary of the results is detailed in Table 3. In the breast milk SC, the genes *IFNA1* and *DCD* yielded Ct values >35 and were not further analyzed whereas *VASP* which was found significantly downregulated by microarray analysis was, however, not confirmed in the total subpopulation of volunteers analyzed by qRT-PCR (median FC = −1.04, considered a no change in this study). On the other hand, our qRT-PCR analyses supported the downregulation of *PLAUR* and *IFNGR1* and the upregulation of *STC1* and *IL19* in the breast milk SC from women with mastitis after treatment with the probiotic. As indicated by the range values [Min, Max], the individual results fluctuated from downregulation to upregulation changes indicating a high variability in the gene expression responses. For a clearer visualization of this variability, Table 3 also displays the distribution of the gene expression changes in % of individuals where the gene was induced, repressed or did not displayed a change together with their corresponding median FC and [Min, Max] ranges. These data evidenced that the average induction detected in *STC1* and *IL19* was confirmed in approximately 60 and 70% of the participants, respectively. On the other hand, the downregulation of *PLAUR* and *IFNGR1* was detected in approximately 65 and 53% of the participants, respectively.

**Table 2:** Validation of the microarrays results for the selected genes in breast milk isolated SC samples (*n* = 5 volunteers) following the intake of the probiotic *L. salivarius* PS2.

**Patient Code**	***PLAUR***			***IFNGR1***			***VASP***			***IL19***			***STC1***		
	**Rt-qPCR**	**Microarrays**		**Rt-qPCR**	**Microarrays**		**Rt-qPCR**	**Microarrays**		**Rt-qPCR**	**Microarrays**		**Rt-qPCR**	**Microarrays**	
		**(5 probes)**	**Median**		**(2 probes)**	**Median**		**(1 probe)**	**Median**		**(2 probes)**	**Median**		**(4 probes)**	**Median**
EM36	↓−1.2	−1.3, −1.3, −1.3, −1.3, +1.1	↓−1.3	↓−1.8	−1.5, −1.5	↓−1.5	↓−1.9	−1.4	↓−1.4	↑+1.9	+1.7, +1.5	↑+1.6	↓−1.8	−1.5, −1.9, −1.5, −1.5	↓−1.5
ML28	↓−2.0	−1.9, −1.5, −1.1, −1.9, −1.8	↓−1.8	↓−1.8	−1.9, −2.6	↓−2.3	↓−1.6	−1.5	↓−1.5	+1.1 (NC)	+1.3, −1.0	+1.1 (NC)	↑+2.4	+1.7, +2.0, +1.7, +1.7	↑+1.7
MM35	↓−1.4	−1.7, −1.5, −1.5, −1.2, +1.0	↓−1.5	↓−1.4	−1.5, −1.2	↓−1.4	↓−1.3	−1.0	−1.0 (NC)	↓−1.5	−1.2, −1.5	↓−1.4	↑+6.6	+5.8, +5.3, +3.1, +4.2	↑+4.8
EM18	↓−2.2	−3.0, −2.8, −1.3, −2.7, −2.4	↓−2.7	↓−1.9	−1.8, −3.5	↓−2.7	↓−2.1	−1.7	↓−1.7	↑+2.3	+2.0, +1.2	↑+1.6	↑+1.7	+1.8, +1.6, +1.4, +1.6	↑+1.6
BM01	↑+1.4	+1.5, +1.4, +1.0, +1.4, +1.2	↑+1.4	↑+1.3	+1.1, +1.7	↑+1.4	↑+1.2	+1.1	+1.1 (NC)	↑+1.2	+1.2, +1.6	↑+1.4	↑+1.3	+1.3, +1.2, +1.9, +1.3	↑+1.7

*Results are presented as FC as calculated by RT-qPCR and by microarrays*.

*For the microarrays results we have indicated the number of probes represented in the array for each gene with their corresponding values and the median value for all the probes. FC-values >+1.2 or < −1.2 were considered a no change, NC*.

**Table 3:** Differential gene expression (by RT-qPCR) in breast milk SC (*n* = 17) and in blood mononuclear cells (*n* = 19) from women with mastitis between day 0 and day 21 (post-intervention) with the probiotic *L. salivarius* PS2.

	**Breast milk SC (*****n*** = **17)**			**Blood mononuclear cells (*****n*** = **19)**		
**Gene**	**Median [Min, Max] (all data)**	% Patients*	**Median [Min, Max]**	**Median [Min, Max] (all data)**	% Patients*	**Median [Min, Max]**
*STC1*	↑+1.4 [−4.1, +6.6]	58.8% ↑	+1.6 [+1.3, +6.6]	Below detection limits		
		17.6% ↓	−1.8 [−4.1, −1.5]			
		23.5% (NC)	−1.1 [−1.1, +1.1]	
*PLAUR*	↓-1.2 [−5.4, +8.5]	23.5% ↑	+3.7 [+1.2, +8.5]	(NC) +1.0 [−2.4, +10.8]	42.1% ↑	+1.7 [+1.2, +10.8]
		64.7% ↓	−2.1 [−5.4, −1.2]		36.8% ↓	−1.5 [−2.4, −1.2]
		11.8% (NC)	−1.1 [−1.1, +1.0]		21.1% (NC)	+1.0 [−1.1, +1.0]
*IFNGR1*	↓-1.4 [-2.6, +3.3]	29.4% ↑	+1.6 [+1.3, +3.3]	(NC) +1.1 [−1.4, 6.3]	42.1% ↑	+1.7 [+1.2, +6.3]
		52.9% ↓	−1.9 [−2.6, −1.4]		10.5% ↓	−1.3 [−1.4, −1.2]
		17.6% (NC)	+1.1 [-1.1, +1.1]		47.3% (NC)	+1.1 [−1.1, +1.1]
*VASP*	(NC) −1.04 [−3.4, +2.3]	23.5% ↑	+2.2 [+1.2, +2.3]	(NC) +1.0 [−2.5, 16.2]	26.3% ↑	+1.5 [+1.3, +16.2]
		47.0% ↓	−1.7 [−3.4, −1.3]		31.6% ↓	−1.4 [−2.5, −1.2]
		29.4% (NC)	1.0 [−1.1, +1.1]		42.1% (NC)	+1.0 [−1.1, +1.1]
*IL19*	↑+1.3 [−1.6, +5.4]	70.5% ↑	+1.6 [+1.2, +5.4]	(NC) +1.0 [−2,567, 10.6]	26.3% ↑	+3.0 [+1.2, +10.6]
		11.8% ↓	−1.5 [−1.6, −1.5]		47.4% ↓	−2.1 [−2,567, 1.5]
		11.8% (NC)	+1.1 [−1.0, +1.1]		26.3% (NC)	+1.1 [+1.0, +1.1]
*IFNA1*	Below detection limits			↓-1.2 [−9.6, 31.8]	36.8% ↑	+1.8 [+1.2, +31.8]
					52.6% ↓	−1.5 [−9.6, −1.2]
					10.5% (NC)	−1.1 [−1.1, −1.1]
*DCD*	Below detection limits			Below detection limits		

Results are presented as FC median values (FC >−1.2 and < +1.2 were considered a no change).*The percentage of patients exhibiting upregulation (↑), downregulation (↓) or no change for each specific gene is also indicated. FC, Fold-change; NC, No change; Range of values, [Min, Max].

We further analyzed the expression changes for the same genes in the leukocytes isolated from the mastitis patients before and after the consumption of the probiotic (Table 3). In this type of cells, *STC1* and *DCD* did not reach the levels of detection and were not further analyzed. The average (median) expression levels of *PLAUR, IFNGR1, VASP*, and *IL19* was unchanged after treatment with the probiotic and the individual results were evenly distributed into upregulation, downregulation and (or) no-change responses. Only *IFNA1* tended to be downregulated in the leukocytes following the intake of the probiotic (~53% of the volunteers). Overall these results display a high interindividual variability in the gene expression responses to the intake of the *L. salivarius* PS2 probiotic both in the milk isolated SC and in the blood isolated leukocytes from women with mastitis. To further illustrate these results and for comparative purposes, we have included Supplementary Tables S6A,B with a list of the gene expression individual changes occurring in the milk SC and the leukocytes, respectively. These tables also display the corresponding individual effects of the probiotic in the bacterial counts, immune cell counts and in the levels of several specific plasma cytokines (Espinosa-Martos et al., 2016). We did not find any significant relationship between the reduction of the bacterial counts or the changes in the immune cell proportions after treatment with the probiotic, with the changes detected in any of the genes analyzed or the circulating cytokines. There was no association either for the gene expression responses occurring between the two types of cells.

## Discussion

Many probiotics such as bifidobacteria and lactobacilli are being widely investigated for their beneficial health effects against inflammatory diseases (Iqbal et al., 2014). The efficacy of some probiotics to promote the regression of the mastitis-associated symptoms and the reduction of the bacterial and somatic cell counts in milk has been already shown in some human studies (Jiménez et al., 2008; Arroyo et al., 2010). Very recently, the human milk isolated probiotic, *L. salivarius* PS2, was also proven to be very effective against mastitis (Espinosa-Martos et al., 2016). Milk samples from women suffering mastitis exhibited higher counts of *Staphylococcus epidermidis, S. aureus*, streptococci and corynebacteria than milk samples from healthy women. The *L. salivarius* PS2 was able to decrease the staphylococcal/streptococcal counts and the patients also experienced a complete or notable improvement of the disease symptoms after 21 days of treatment (Espinosa-Martos et al., 2016). Further, this strain led to substantial changes in some immune cell subpopulations as well as in the levels of several immunoglobulins, cytokines, and chemokines (e.g., IL8, IL7) but a large interindividual variability was noted (Espinosa-Martos et al., 2016).

It has been hypothesized that the probiotics may exert these benefits against mastitis by competing for the habitat, producing biological compounds that inhibit the pathogen growth, and (or) stimulating the host immune defenses (Fernández et al., 2014). Nevertheless, the actual molecular mechanisms underlying the infection process as well as the effects of these probiotics against it are not yet fully understood. The establishment of the molecular pathways associated with the infection process in mastitis as well as of disease-associated genes as biomarkers to aid in the detection of mastitis and to monitor the response to therapy has been investigated mostly *in vitro* and in farm animals. The biological complexity of this disease has prompted the use of “omics” techniques and system biology approaches to investigate global transcription changes in the animal mammary gland tissue during mastitis infection with different pathogens (Swanson et al., 2009; de Greeff et al., 2013; Sipka et al., 2014; Sun et al., 2015). The use of RNA extracted from breast milk samples was shown to be a safe, non-invasive and useful means to investigate the human mammary gland transcriptome (Boutinaud et al., 2015). This method was recently applied to explore the gene expression profiles of human breast milk cells associated with the lactation cycle, involution and mastitis infection using microarrays and quantitative RT-qPCR (Sharp et al., 2016). To the best of our knowledge, our study is the first to apply microarrays and quantitative RT-qPCR to the study of the gene expression changes occurring in human milk cells from women suffering infectious mastitis following intervention with a probiotic.

Transcriptomic analysis of *in vitro Staphylococcus aureu*s*-*infected bovine macrophages (Lewandowska-Sabat et al., 2013) and bovine epithelial cells (Wang et al., 2013) showed that the infected cells displayed the activation of genes involved in the inflammatory response, cytokine signaling pathways, metabolism and apoptosis suggesting that both types of cells may be implicated in the mastitis infection process of the mammary gland. The application of these holistic studies to mastitis infection in animals has been extensively reviewed (Loor et al., 2011). The authors brought together some of the main biological functions and pathways that appear to be altered in various animals during the mastitis infection caused by different pathogens and showed that some of them were repeatedly found to be involved in mastitis, ranging from very broad categories such as “immune response” and “lipid/energy metabolism” to more specific canonical and signaling pathways including: “acute phase response signaling,” “chemokine signaling pathway,” “cytokine-cytokine receptor interaction,” “NOD-like receptor signaling pathway,” “Toll-like receptor signaling pathway,” “RIG-like receptor” “natural killer cell mediated cytotoxicity,” “complement and coagulation cascades” or “fatty acid metabolism.” In good agreement with some of the previous results and also with those published by Sharp et al. (2016) in women, our functional analysis also detected the regulation of the inflammatory response with the involvement of immune cells such as the T-lymphocytes and chemokine signaling pathways as well as cell processes related with cell survival (cell proliferation, cell angiogenesis) (Supplementary Table S5) further supporting the contribution of these processes in the mastitis infection in women.

With regards to the specific molecules that might be involved in the mastitis process, differential gene expression between the mammary gland infected with *Streptococcus uberis* and control tissue in heifers (Swanson et al., 2009; de Greeff et al., 2013) evidenced the involvement of many genes implicated in diverse functions: Toll-like receptors (*TLR2*), ficolins and lipolysaccharide binding protein (*LBP*) in pathogen recognition, haptoglobin (*HP*) in acute phase response, superoxide dismutase (*SOD*) and indoleamine 2,3-dioxygenase 1 (*IDO1*) in oxidative stress and bactericidal activity, calgranulins or S100 calcium-binding proteins (*S100A2, S100A8, S100A9*), chemokines (*CXCL10, CXCL6, CCL8, CCL2*), interleukins and interleukin-related molecules (*IL18, IL6R, IL10R*), interferon regulatory factors (*IRF1*), interferon induced proteins (*IFIT4, IFIT5*) in the immune/inflammatory response and chemoattraction of neutrophils, plasminogen activator, urokinase receptor (*PLAUR*) in tissue repair mechanisms, or SON DNA Binding Protein (*SON*) in cell death and cell proliferation, to mention only a few examples. Also, in a recent meta-analysis of microarrays-based transcriptomic studies of the bovine mammary gland with *E. coli* induced-mastitis, a number of meta-genes have been identified as potential biomarkers of the disease (Sharifi et al., 2018). The top list of genes include: several pro-inflammatory cytokines (*CXCL2, CXCL8* (*IL8*), *CXCL1, CCL20*), calcium binding proteins (*S100A9, S100A8*), the complement factor B (*CFB*), phosphodiesterase 4B (*PDE4B*), caspase 4 (*CASP4*), *HP* or the zinc finger CCCH-type containing 12A (*ZC3H12A*). In women (Sharp et al., 2016), a list of genes were also reported to be up- or downregulated in mastitis in comparison with healthy lactation. Some of these genes had also been previously detected in the animal studies: e.g., interferon-induced transmembrane proteins (*IFITM2, IFITM1*), *S100A8, IDO1, PLAUR*, some interleukins and chemokines (*CXCL10, CCL8*). In addition, in this study, predicted activation status of transcription regulators showed the activation of *STAT1, MITF*, and various interferon regulatory factors in mastitis milk. Overall, and regarding specific genes with different expression levels in the samples from mastitis and from healthy subjects, we found little agreement with the significant differentially expressed individual genes detected in our study. We did detect, however, a moderate but significant upregulation of *ZC3H12A* (FC = +1.2, *p* = 0.039) when comparing mastitis vs. healthy human samples (Supplementary Table S3). This meta-gene that has been identified as a potential biomarker for *E. coli* mastitis with a high accuracy (~84%) (Sharifi et al., 2018) encodes for a monocyte chemotactic protein-induced protein 1 (MCP-1) critically involved in inflammatory responses and has an important positive regulatory activity on interferon antiviral activity by promoting type-1 interferons signaling (Qian et al., 2018). It is worth mentioning that after a second revision of our original microarray data, we found some agreement for specific genes, i.e., induction of several members of the interferon induced transmembrane protein family, *IFITM1*, and *IFITM2*, of the calcium-binding proteins *S100A7, S100A12* or *S100P* and of the transcription factors, *STAT1, IRF1, IRF7*, and *MITF* although these results were not significant and had not surpassed our statistical cut-off criteria (*p* < 0.05). The reduced number of microarrays performed in the studies revised here, e.g., *n* = 1 (lactation 24) vs. *n* = 1 (mastitis 23) (Sharp et al., 2016) or, in our own study, *n* = 7 (mastitis) vs. *n* = 3 (healthy) together with the heterogeneous composition of the samples analyzed greatly contribute to the high inter-sample (inter-individual) variability of the results and the lack of power to detect more significant commonly responsive genes.

Nevertheless, the involvement of immune cells (lymphocytes, macrophages, neutrophils) which are involved in protecting the gland from infection and the triggering of inflammatory responses through chemotactic pathways in the mammary gland during mastitis seem to be well established (Sharp et al., 2016) and some of the genes found to be associated with these functions in animal and human studies might constitute potential targets involved in the infection process and, possibly, targets of the response to therapy against mastitis. Along these lines, the effect of an antibiotic (cefapirin) alone or combined with prednisolone on gene expression profiles in bovine with *E. coli* mastitis evidenced for the first time the downregulation in the mammary gland tissue of the expression levels of chemokines such as *CXCL1, CXCL2*, and *CXCL8* (*IL8*) in response to the treatment (Sipka et al., 2014).

Based on all the previously published results, some molecular targets affected in mastitis and potentially responsive to treatment might be some of the genes related with the interferon-mediated immune response, chemokines and cytokines, and genes related with cell survival (proliferation, adhesion, angiogenesis). In our study, we selected a number of genes that exhibited a significant average response in the milk SC to the treatment with the probiotic *L. salivarius* PS2 and that were involved in some of these biological processes (Table 1). For instance, *PLAUR* is involved in the regulation of inflammatory processes and has been found upregulated in the mammary gland cells infected with mastitis both in animals (de Greeff et al., 2013) and in humans (Sharp et al., 2016). Our microarray analysis showed that the overall expression of this gene was significantly downregulated in the milk SC samples (average FC = −2.0, *p* = 0.0399, Table 1) after treatment with the probiotic. RT-qPCR confirmed the average downregulation of this gene (FC = −1.2) but it also showed that the downregulation was observed in ~65% of the participants (FC = −2.1) whereas the remaining participants exhibited either upregulation (23.5%) or no change (11.8%) (Table 3). In a similar manner, the downregulation of *IFNGR1* and the upregulation of *IL19* and *STC1* detected by our microarray analysis were also confirmed by RT-qPCR in approximately 53, 70, and 59% of the sample population, respectively (Table 3). These results give preliminary evidence of these genes as genes associated with mastitis infection and potentially responsive to the treatment with the probiotic *L. salivarium* in breast milk isolated SC. In addition, the results also display the interindividual variability in the response with some individuals exhibiting upregulation, downregulation or no change in the expression levels.

Peripheral blood isolated immune cells, mostly mononuclear lymphocytes and monocytes, have been widely used in nutrigenomic studies because of their easy availability and also, because they have been considered to reflect the effects of dietary interventions at the levels of gene expression. Nevertheless, nutrigenomic studies of these cells from human intervention trials have not yet confirmed them as a surrogate tissue and (or) to provide candidate gene targets responsive to a therapy (de Mello et al., 2012) and further studies are still needed. One additional objective of our study was to find out whether there were any potential common responses or relationship for specific responsive genes between the milk SC and the isolated blood leukocytes following treatment with the probiotic. Previous studies had measured gene expression responses in milk SC and in blood immune cells in response to infection with *Staphylococcus aureus* in cow (Tao and Mallard, 2007) and in goat (Cremonesi et al., 2012) and showed that despite some specific similarities such as the induction of the gene pentatrexin 3 (*PTX3*) involved in the regulation of innate resistance to pathogens, there were not many other coincidences in the gene response in both type of cells. In agreement with this, we did not find any significant common responses for the specific genes investigated between the milk SC and the leukocytes or any association between their expression profiles which support the notion that peripheral blood immune cells may not reflect the changes occurring in the breast milk SC. Nevertheless, future studies are needed to further confirm this. We also detected a large interindividual variability in the gene responses in the circulating immune cells. Overall, our results evidence the complexity and difficulties associated with the search and detection of suitable genes as molecular biomarkers of response to any particular treatment. This is especially hampered by the large gene expression interindividual variability in humans which is affected by (1) methodological aspects inherent to the experimental protocols and (2) human intrinsic differences. Along these lines, gene expression has been reported to be gene- and tissue-specific and enhanced in disease-associated genes as well as by the protocols (Nú-ez-Sánchez et al., 2017). In order to improve the selection and evaluation of responsive gene targets, the methodological variability must be controlled by means of improving sample attaining, description, processing and analyses protocols (Pokimica and García-Conesa, 2018). On the other hand the factors inherent to humans and causing interindividual variability are many (sex, age, health status, genetic make-up, microbiota) and we are only beginning to understand them (Manach et al., 2017). Future studies should be designed to investigate the effects of these factors on gene expression variability.

## Conclusion

Despite the limitations of this study, small number of participants and high interindividual variability, our analysis further supports the potentiality of using breast milk SC isolated from women with mastitis and gene expression profiling as a means to explore molecular changes in response to treatment with probiotics. Our results indicate that following the intake of *L salivarius* PS2 for 21 days, there are changes at the molecular level in the SC that reflect alterations in inflammatory and cell-growth related signaling pathways and in specific genes (*PLAUR, IL19, STC1, IFNGR1*) which may be potentially responsive targets to the treatment. Our study also shows a lack of association between the observed molecular responses in the SC and those occurring in the circulating leukocytes. In both types of samples we observed large interindividual variability in the gene responses. Our study also reinforces the need to continue improving the design of future human intervention trials to better evaluate and select suitable responsive molecular targets. In addition to increasing the number of individuals/samples analyzed, the improvement of the experimental protocols and of the analysis and presentation of gene expression changes are also necessary. Further, assessing the interindividual variability for each gene and tissue sample and specifying the distribution of the results into responders (upregulation and downregulation) and non-responders (no change) should be implemented. The inclusion of all and each of the individual participant results may be of benefit. Implementing these practices in future clinical studies will be relevant for integrative meta-analyses that will contribute to a better understanding of gene expression responses and the search and discovery of potential targets to therapy.

## Author contributions

EJ, JR, and M-TG-C conceived the study and designed the experiments. JA, EJ, IE-M, and M-TG-C performed the experiments, analyzed, and interpreted the results. All authors contributed to the writing of the manuscript and approved it.

### Conflict of interest statement

The authors declare that the research was conducted in the absence of any commercial or financial relationships that could be construed as a potential conflict of interest.
