# Supplementary material for: An Exploratory Search for Potential Molecular Targets Responsive to the Probiotic Lactobacillus salivarius PS2 in Women With Mastitis: Gene Expression Profiling vs. Interindividual Variability
Source: Front Microbiol. 2018 Sep 13;9:2166. doi: 10.3389/fmicb.2018.02166 (PMC6146105; doi:10.3389/fmicb.2018.02166)
Supplement: Supplementary file 1 [file Table_1.DOCX]

**Supplementary Table S1.-** Demographic data of the women participating in the study.

|  | Mastitis (n = 23) | | Healthy (n = 8) | |  |
| --- | --- | --- | --- | --- | --- |
|  | Median | IQR | Median | IQR | *p*-value (KW) |
| Feeding Days | 49.5 | 30 - 76.50 | 107.5 | 88 - 153.75 | 0.004 |
| Mother's age | 33 | 31.75 - 35.25 | 36 | 33.75 - 36.50 | 0.131 |
| Sibling's number | 1 | 1.00 - 2.00 | 2 | 1.75 - 2.00 | 0.038 |
|  | Frequency | (%) | Frequency | (%) | *p*-value |
| Delivery type |  |  |  |  |  |
| C-section | 4 | 16,7 | 0 | 0,0 | 0.993 |
| Vaginal | 20 | 83,3 | 8 | 100,0 |  |
| Antibiotic treatment |  |  |  |  |  |
| Yes | 8 | 33,3 | 1 | 12,5 | 0.277 |
| No | 16 | 66,7 | 7 | 87,5 |  |
| Feeding days |  |  |  |  |  |
| 18 - 46 | 11 | 45,8 | 0 | 0,0 | 0.029 |
| 46 - 90 | 8 | 33,3 | 3 | 37,5 |  |
| 90 - 450 | 5 | 20,8 | 5 | 62,5 |  |
| Mother's age |  |  |  |  |  |
| 27 - 32.3 | 10 | 41,7 | 1 | 12,5 | 0.130 |
| 32.3 - 35.7 | 8 | 33,3 | 2 | 25,0 |  |
| 35.7 - 41 | 6 | 25,0 | 5 | 62,5 |  |
| Siblings |  |  |  |  |  |
| 1 | 17 | 70,8 | 2 | 25,0 | 0.085 |
| > 1 | 7 | 29,2 | 6 | 75,0 |  |
