# Supplementary material for: An Exploratory Search for Potential Molecular Targets Responsive to the Probiotic Lactobacillus salivarius PS2 in Women With Mastitis: Gene Expression Profiling vs. Interindividual Variability
Source: Front Microbiol. 2018 Sep 13;9:2166. doi: 10.3389/fmicb.2018.02166 (PMC6146105; doi:10.3389/fmicb.2018.02166)
Supplement: Supplementary file 2 [file Table_2.DOC]

**Supplementary Table S2.-** Genes selected for qRT-PCR analyses and specific Applied Biosystems (ABI) TaqMan assays employed.

| ABI Assay ID | RefSeq | Approved^a^  gene symbol | Approved^a^  gene name |
| --- | --- | --- | --- |
| Hs00174970_m1 | NM_003155.2 | *STC1* | Stanniocalcin 1 |
| Hs00958880_m1 | NM_001005376  NM_001005377  NM_002659 | *PLAUR* | Plasminogen activator, urokinase receptor |
| Hs00988304_m1 | NM_000416.2 | *IFNGR1* | Interferon gamma receptor 1 |
| Hs01100128_m1 | NM_003370.3 | *VASP* | Vasodilator-stimulated phosphoprotein |
| Hs00604657_m1 | NM_153758.2 | *IL19* | Interleukin 19 |
| Hs04190680_gH | NM_006900.3  NM_024013.2 | *IFNA1 IFNA13* | Interferon alpha-1/13 |
| Hs00364976_m1 | NM_053283.2 | *DCD* | Dermcidin |
| *Reference genes* | | | |
| Hs02758991_g1 | NM_001256799.1  NM_002046.4 | *GAPDH* | Glyceraldehyde-3-Phosphate Dehydrogenase |
| Hs00427620_m1 | NM_001172085.1  NM_003194.4 | *TBP* | TATA box binding protein |
| Hs00172187_m1 | NM_000937.4 | *POLR2A* | Polymerase (RNA) II Subunit A |

^a^HGNC, Hugo Gene Nomenclature Committee.
