# Supplementary material for: An Exploratory Search for Potential Molecular Targets Responsive to the Probiotic Lactobacillus salivarius PS2 in Women With Mastitis: Gene Expression Profiling vs. Interindividual Variability
Source: Front Microbiol. 2018 Sep 13;9:2166. doi: 10.3389/fmicb.2018.02166 (PMC6146105; doi:10.3389/fmicb.2018.02166)
Supplement: Supplementary file 5 [file Table_5.DOCX]

**Supplementary Table S5.-** Functional analysis: summary of top cellular and molecular functions (by Gene Ontology, GO, terms analysis) and pathways (by Partek Genomic Suite analysis) enriched in differentially express (DE) in participants with mastitis *vs* healthy women, and in women with mastitis before (day 0) and after (day21) the treatment with *Lactobacillus salivarius* PS2.

| Probes upregulated  (FC >+1.2)  and downregulated (FC <-1.2)  (*p*-value <0.05) |  | Mastitis *vs.* Healthy (day 0) | Mastitis (day 21) *vs.* Mastitis (day 0) |
| --- | --- | --- | --- |
|  | GO terms analysis using DAVID  (*p*-value<0.05) | *Cluster 1*  *(Donwregulated, 1068 probes)*  RNA splicing, transport and localization  Purine ribonucleoside monophosphate biosynthetic process  Glutamine metabolic process  Membrane fusion, protein targeting to membrane, inner mitochondrial membrane organization  Mitotic spindle organization | *Cluster 1*  *(Downregulated, 81 probes)*  Positive regulation of cell death, induction of apoptosis  Regulation of transcription |
|  |  | *Cluster 2*  *(Upregulated, 309 probes)*  Immune response  Lymphocyte, T-cell, natural killer cell activation, positive regulation of alpha-beta T cell differentiation, regulation of defense response to virus  Response to nutrient levels and extracellular stimulus  Regulation of transcription and translation  Transmembrane receptor protein tyrosine kinase signaling pathway  Positive regulation of nucleobase, nucleoside, nucleotide and nucleic acid metabolic and phosphorylation processes  Regulation of cell proliferation, cell adhesion and angiogenesis | *Cluster 2*  *(Upregulated, 387 probes)*  Amino acid catabolic process, glycine metabolic process  GPI anchor biosynthetic process, phosphoinositide metabolic process  Transmembrane transport, dicarboxylic acid transport  Morphogenesis |
|  | Pathways analysis using  Partkek Genomics Suite  (uppermost altered inflammation and cell growth/survival related pathways) | Systemic lupus erythematosus  PI3K-Akt signaling pathway  Focal adhesion  Rap1 signaling pathway  Natural killer cell mediated cytotoxicity  Chemokine signaling pathway  Herpes simplex infection  Cytokine-cytokine receptor interaction | PI3K-Akt signaling pathway  Systemic lupus erythematosus  Cytokine-cytokine receptor interaction  Influenza A  Jak-STAT signaling pathway  Tuberculosis  Natural killer cell mediated cytotoxicity  Herpes simplex infection  Focal adhesion  Rap1 signaling pathway  Toll-like receptor signaling pathway  Leukocyte trans-endothelial migration |

**Abbreviations:** GPI, glycosylphosphatidylinositol ; PI3K, Phosphatidylinositol-4,5-Bisphosphate 3-Kinase Catalytic Subunit Delta; Akt, AKT Serine/Threonine Kinase 1; Jak, Janus Kinase; STAT, Signal Transducer And Activator Of Transcription; Rap1, RAP1A, Member Of RAS Oncogene Family.
