# Supplementary material for: An Exploratory Search for Potential Molecular Targets Responsive to the Probiotic Lactobacillus salivarius PS2 in Women With Mastitis: Gene Expression Profiling vs. Interindividual Variability
Source: Front Microbiol. 2018 Sep 13;9:2166. doi: 10.3389/fmicb.2018.02166 (PMC6146105; doi:10.3389/fmicb.2018.02166)
Supplement: Supplementary file 6 [file Table_6.DOCX]

**Supplementary Table S6a.-** Interindividual variability in the changes of the expression levels of selected genes in breast milk isolated SC samples following the intake of the probiotic *L. salivarius* PS2. For comparative purposes, the effects of the probiotic in bacterial counts, blood cell counts and various protein targets for each individual is included*.

| Patient  Code*  (n=22) | Total bacterial counts | % Neutrophils | % Macrophages | Gene expression changes (FC)^1^ | | | | | Cytokines changes (FC) | | | | |
| --- | --- | --- | --- | --- | --- | --- | --- | --- | --- | --- | --- | --- | --- |
|  |  |  |  | *PLAUR* | *IFNGR1* | *VASP* | *IL19* | *STC1* | TNF | IL8 | IL6 | IL7 | IFNγ |
| EM36 | ↓-39% | ↓-82% | ↑+38% | ↓-1.2 | ↓-1.8 | ↓-1.9 | ↑+1.9 | ↓-1.8 | ↓ | ↑+1.7 | N.D.^2^ | ↓ | N.D. |
| NZ38 | ↓-23% | ↓-58% | ↑+42% | ↓-3.1 | ↓-2.4 | N.C. +1.1 | ↑+1.7 | N.C. -1.1 | ↑ | ↑+8.2 | ↑ | ↑+3.0 | ↑ |
| IB39 | ↓-20% | ↓-69% | ↑+6% | ↓-5.4 | ↑+1.4 | ↓-3.4 | ↑+5.4 | ↑+3.5 | ↓ | ↓-50.0 | ↓ | ↓-3.0 | ↓ |
| FC41 | - | - | - | ↑+1.2 | N.C. +1.1 | N.C. -1.0 | ↑+1.4 | N.C. +1.1 | - | - | - | - | - |
| LB27 | ↓-9% | ↓-62% | ↑+43% | ↓-1.2 | ↑+2.5 | ↑+2.2 | ↑+1.4 | ↓-4.1 | ↑ | ↓-1.7 | ↑ | ↑+3.0 | N.D. |
| ML28 | ↓-21% | ↓-83% | ↑+44% | ↓-2.0 | ↓-1.8 | ↓-1.6 | N.C. +1.1 | ↑+2.4 | ↓-3.0 | ↑+2.2 | N.D. | N.C. +1.0 | N.D. |
| SM31 | ↓-24% | ↓-42% | ↑+21% | ↓-1.4 | ↑+1.6 | N.C. -1.1 | ND | ↑+4.8 | ↑ | ↑+1.4 | N.C. +1.0 | ↓-3.0 | N.D. |
| MM35 | ↓-38% | ↓-48% | ↑+62% | ↓-1.4 | ↓-1.4 | ↓-1.3 | ↓-1.5 | ↑+6.6 | N.D. | N.C. -1.1 | N.D. | ↓ | N.D. |
| EL16 | ↓-27% | ↓-67% | ↑+48% | N.C. +1.0 | N.C. +1.1 | ↓-1.3 | ↓-1.6 | ↑+1.5 | ↑ | N.C. -1.1 | N.C. -1.0 | N.C. -1.1 | N.D. |
| EM18 | ↓-25% | ↓-82% | ↑+3% | ↓-2.2 | ↓-1.9 | ↓-2.1 | ↑+2.3 | ↑+1.7 | ↓ | ↓-5.6 | ↓-3.4 | ↑ | N.D. |
| LC20 | ↓-29% | ↓-67% | ↑+61% | ↓-3.0 | ↓-1.8 | ↓-1.7 | ↑+1.5 | ↓-1.5 | N.D. | ↓-1.2 | ↓ | ↓ | N.D. |
| FM25 | ↓-36% | ↓-15% | ↑+22% | ↑+3.3 | N.C. -1.1 | ↑+2.0 | ↑+1.2 | ↑+1.4 | ↑+3.5 | ↑+2.9 | ↓ | ↑+1.5 | N.D. |
| BM01 | ↓-16% | ↑+8% | ↑+7% | ↑+1.4 | ↑+1.3 | ↑+1.2 | ↑+1.2 | ↑+1.3 | ↑ | ↑+1.3 | ↑+10.5 | ↑+539.0 | N.D. |
| AL02 | ↓-23% | ↓-39% | ↑+21% | ↓-1.2 | ↓-2.6 | N.C. +1.0 | ↑+1.2 | NC -1.1 | ↑ | N.C. +1.1 | ↓-2.0 | ↑+7.4 | ↑+3.7 |
| MA03 | ↓-21% | ↑+1% | ↑+9% | N.C. -1.1 | ↓-2.1 | N.C. +1.0 | NC -1.0 | ↑+1.6 | ↑ | ↑+1.5 | ↓-8.1 | ↓-1.7 | N.D. |
| ER11 | ↓-26% | ↓-75% | ↑+20% | ↓-2.3 | ↓-2.4 | ↓-1.3 | ↑+2.0 | ↑+1.4 | ↑ | ↓-2.0 | ↓-6.4 | ↓ | N.D. |
| HW12 | ↓-27% | ↓-67% | ↑+23% | ↑+8.5 | ↑+3.3 | ↑+2.3 | ↑+1.3 | N.C. +1.0 | ↓ | ↓-2.0 | ↑+1.2 | N.D. | N.D. |

^1^: FC-values >+1.2 or <-1.2 were considered a no change (N.C.); ^2^: Not detected (N.D.).*Values obtained from previous publication (Espinosa-Martos et al., 2016).
