# Supplementary material for: An Exploratory Search for Potential Molecular Targets Responsive to the Probiotic Lactobacillus salivarius PS2 in Women With Mastitis: Gene Expression Profiling vs. Interindividual Variability
Source: Front Microbiol. 2018 Sep 13;9:2166. doi: 10.3389/fmicb.2018.02166 (PMC6146105; doi:10.3389/fmicb.2018.02166)
Supplement: Supplementary file 7 [file Table_7.DOCX]

**Supplementary Table S6b.-** Interindividual variability in the changes of the expression levels of selected genes in blood isolated leukocytes following the intake of the probiotic *L. salivarius* PS2. For comparative purposes, the effects of the probiotic in blood cell counts and various protein targets for each individual is included*.

| Patient  Code*  (n=19) | % Lymphocytes | %  Monocytes | %  Mononuclear cells | Gene expression changes by RT-qPCR (FC)^1^ | | | | | Cytokines changes | | | | |
| --- | --- | --- | --- | --- | --- | --- | --- | --- | --- | --- | --- | --- | --- |
|  |  |  |  | *PLAUR* | *IFNGR1* | *VASP* | *IL19* | *IFNA1* | TNF | IL8 | IL6 | IL7 | IFNγ |
| EM36 | ↓-24% | ↑+5% | ↑+23% | ↓-1.5 | N.C. +1.1 | N.C. -1.0 | N.C. +1.1 | ↓-1.2 | ↑+1.5 | ↑+1.5 | ↑+3.1 | ↑+2.1 | ↑+2.3 |
| NZ38 | ↓-8% | ↓-7% | ↑+5% | ↑+1.4 | N.C. +1.1 | ↑+1.3 | ↓-2.2 | ↓-1.2 | ↓-1.9 | N.C. -1.1 | ↓-1.2 | N.C. -1.1 | ↓-1.6 |
| IB39 | ↓-19% | ↓-11% | ↑+1% | ↑+1.2 | ↑+1.2 | ↑+1.5 | N.C. +1.1 | ↓-1.3 | ↑+1.8 | N.C. -1.1 | ↑+1.4 | ↑+1.3 | ↑+1.4 |
| FC41 | N.D. | N.D. | N.D. | ↑+2.9 | ↑+1.2 | N.C. +1.0 | N.C. +1.1 | ↑+1.2 | N.D. | N.D. | N.D. | N.D. | N.D. |
| LB27 | ↓-14% | ↓-16% | ↑+12% | ↑+1.3 | ↑+1.6 | N.C. +1.1 | ↓-1.8 | ↓-1.4 | N.C. -1.1 | N.C. +1.1 | N.C. +1.0 | ↓-1.4 | ↑+1.2 |
| ML28 | ↑+5% | ↓-4% | ↓-3% | ↓-1.2 | N.C. -1.0 | N.C. +1.0 | ↓-2.1 | ↑+1.8 | ↓-2.3 | N.C. +1.1 | ↓-1.5 | ↓-1.3 | ↓-1.4 |
| SM31 | ↓-13% | ↑+43% | N.C. | ↓-1.5 | N.C. +1.1 | ↓-1.2 | ↓-1.9 | N.C. -1.1 | ↓-1.2 | N.C. -1.1 | N.C. +1.1 | N.C. +1.0 | ↓-1.8 |
| MM35 | ↑+3% | ↑+2% | ↑+2% | ↓-1.3 | N.C. -1.1 | N.C. +1.1 | N.C. +1.1 | ↓-1.7 | ↑+1.4 | N.C. +1.0 | ↑+1.5 | ↑+1.5 | ↑+1.2 |
| EM18 | ↑+32% | ↑+10% | ↓-13% | N.C. +1.0 | ↑+1.5 | ↑+1.4 | N.C. +1.0 | ↑+1.5 | ↓-1.6 | N.C. +1.1 | N.D. | N.D. | ↑+1.3 |
| LC20 | ↓-23% | ↓-22% | ↑+21% | N.C. +1.0 | ↓-1.4 | N.C. -1.1 | ↓-2.8 | ↓-4.1 | ↓-1.4 | ↓ | ↑ | N.D. | ↓-2.6 |
| FM25 | ↑+18% | ↑+14% | ↓-3% | ↓-1.5 | N.C. -1.1 | ↓-1.3 | ↑+1.6 | ↑+2.0 | ↓-1.8 | N.C. -1.1 | ↓-1.3 | ↑+1.4 | N.C. -1.1 |
| BM01 | ↓-3% | ↑+39% | ↑+3% | ↑+1.2 | N.C. +1.1 | ↓-1.5 | ↓-1.7 | ↑+1.5 | ↑ | ↓-1.3 | N.D. | N.D. | ↓ |
| AL02 | ↓-4% | ↓-8% | ↑+2% | ↑+3.2 | ↑+1.7 | N.C. +1.1 | ↑+3.9 | ↓-1.9 | ↑+3.1 | ↑+2.0 | N.D. | N.D. | ↑+1.5 |
| HW12 | ↓-1% | ↑+8% | ↓-3% | N.C. -1.1 | N.C. +1.1 | ↓-1.5 | ↑+1.2 | ↓-1.3 | ↓-1.2 | ↓ | N.D. | N.D. | ↓ |
| CM04 | ↓-7% | ↑+20% | ↑+2% | ↓-2.2 | N.C. +1.1 | ↓-2.5 | ↑+3.0 | ↑+31.8 | ↓ | ↓ | N.D. | N.D. | ↓ |
| EG09 | ↓-3% | ↓-9% | ↑+3% | N.C. -1.1 | ↑+2.3 | N.C. -1.1 | ↓-1.5 | N.C. -1.1 | ↑+3.3 | ↑ | N.D. | N.D. | ↑+1.7 |
| MR06 | ↑+8% | ↓-13% | ↓-4% | ↑+10.8 | ↑+6.3 | ↑+16.2 | ↓-2567 | ↑+31.5 | N.C. +1.0 | ↓-2.5 | N.D. | ↓ | ↓-1.4 |
| TP32 | ↓-26% | ↓-4% | ↑+18% | ↑+1.9 | ↑+1.9 | ↑+1.9 | ↓-3.9 | ↓-2.1 | ↑+1.2 | ↑+1.3 | ↑+1.3 | ↓-1.2 | N.C. +1.0 |
| UP08 | N.D. | N.D. | N.D. | ↓-2.4 | ↓-1.2 | ↓-1.3 | ↑+10.6 | ↓-9.6 | N.D. | N.D. | N.D. | N.D. | N.D. |

^1^: FC-values >+1.2 or <-1.2 were considered a no change (N.C.); ^2^: Not detected or Not determined (N.D.) *Values obtained from previous publication (Espinosa-Martos et al., 2016).
