# Supplementary material for: An Exploratory Search for Potential Molecular Targets Responsive to the Probiotic Lactobacillus salivarius PS2 in Women With Mastitis: Gene Expression Profiling vs. Interindividual Variability
Source: Front Microbiol. 2018 Sep 13;9:2166. doi: 10.3389/fmicb.2018.02166 (PMC6146105; doi:10.3389/fmicb.2018.02166)
Supplement: Supplementary file 8 [file Table_8.DOCX]

Supplementary Material

**An exploratory search for potential molecular targets responsive to the probiotic *Lactobacillus salivarius* PS2 in women with mastitis: gene expression profiling *vs* interindividual variability.**

Javier de Andrés, Esther Jiménez, Irene Espinosa-Martos, Juan Miguel Rodríguez, María-Teresa García-Conesa*

*** Correspondence:**

María-Teresa García-Conesa: [mtconesa@cebas.csic.es](mailto:mtconesa@cebas.csic.es)

**Supplementary Material Legend**

**Supplementary Table S1.-** Demographic data of the women participating in the study.

**Supplementary Table S2.-** Genes selected for qRT-PCR analyses and specific Applied Biosystems (ABI) TaqMan assays employed.

**Supplementary Table S3.-** Gene expression differences between breast-milk somatic cells (SC) from women with mastitis (n=7) and from healthy women (n=3) (Time 0: initial). Selection criteria: *p*-value < 0.05 and Fold-Changes (<-1.2 and > 1.2). Total probes: 1377. Probes upregulated: 309. Probes downregulated: 1068 (see attached Excel file).

**Supplementary Table S4.-** Gene expression differences between breast-milk somatic cells (SC) from women with mastitis and treated with a probiotic strain (n=7). Time 21 days (final) vs. Time 0 (initial). Selection criteria: p-value < 0.05 and Fold-Changes (<-1.2 and > 1.2). Total probes: 468. Probes upregulated: 387. Probes downregulated: 81(see attached Excel file).

**Supplementary Table S5.-** Functional analysis: summary of top cellular and molecular functions (by Gene Ontology, GO, terms analysis) and pathways (by Partek Genomic Suite analysis) enriched in differentially express (DE) in participants with mastitis *vs* healthy women, and in women with mastitis before (day 0) and after (day21) the treatment with *Lactobacillus salivarius* PS2.

**Supplementary Table S6a.-** Interindividual variability in the changes of the expression levels of selected genes in breast milk isolated SC samples following the intake of the probiotic *L. salivarius* PS2. For comparative purposes, the effects of the probiotic in bacterial counts, blood cell counts and various protein targets for each individual is included*.

**Supplementary Table S6b.-** Interindividual variability in the changes of the expression levels of selected genes in blood isolated leukocytes following the intake of the probiotic *L. salivarius* PS2. For comparative purposes, the effects of the probiotic in blood cell counts and various protein targets for each individual is included*.

**
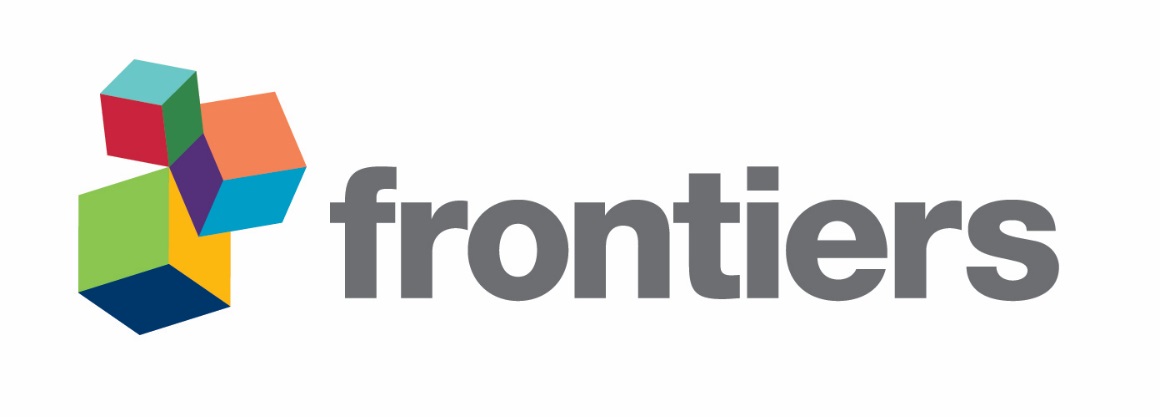
**
